# Supplementary material for: Correlative single molecule lattice light sheet imaging reveals the dynamic relationship between nucleosomes and the local chromatin environment
Source: Nat Commun. 2024 May 16;15:4178. doi: 10.1038/s41467-024-48562-0 (PMC11099156; doi:10.1038/s41467-024-48562-0)
Supplement: Supplementary file 1 — Supplementary Information [file 41467_2024_48562_MOESM1_ESM.pdf]

Supplementary Information for

Correlative single molecule lattice light sheet imaging reveals the dynamic relationship between nucleosomes and the local chromatin environment

Timothy A. Daugird[1, ‡], Yu Shi[2, ‡], Katie L. Holland[3,] Hosein Rostamian[4,5], Zhe Liu[3], Luke D. Lavis[3], Joseph Rodriguez[6], Brian D. Strahl[4,5], Wesley R. Legant[1,2*]

1) Department of Pharmacology, University of North Carolina at Chapel Hill, Chapel Hill, NC, USA
2) Joint Department of Biomedical Engineering, University of North Carolina at Chapel Hill, North Carolina State University, Chapel Hill, NC, USA
3) Janelia Research Campus, Howard Hughes Medical Institute, Ashburn, VA 20147, USA
4) Department of Biochemistry and Biophysics, University of North Carolina at Chapel Hill, Chapel Hill, NC, USA.
5) Curriculum in Genetics and Molecular Biology, University of North Carolina at Chapel Hill, Chapel Hill, NC, USA.
6) National Institute of Environmental Health Sciences, Durham, North Carolina 27709, USA.

‡ - equal contribution, * to whom correspondence should be addressed: legantw@email.unc.edu

This material includes:

- Supplementary Figures 1-16

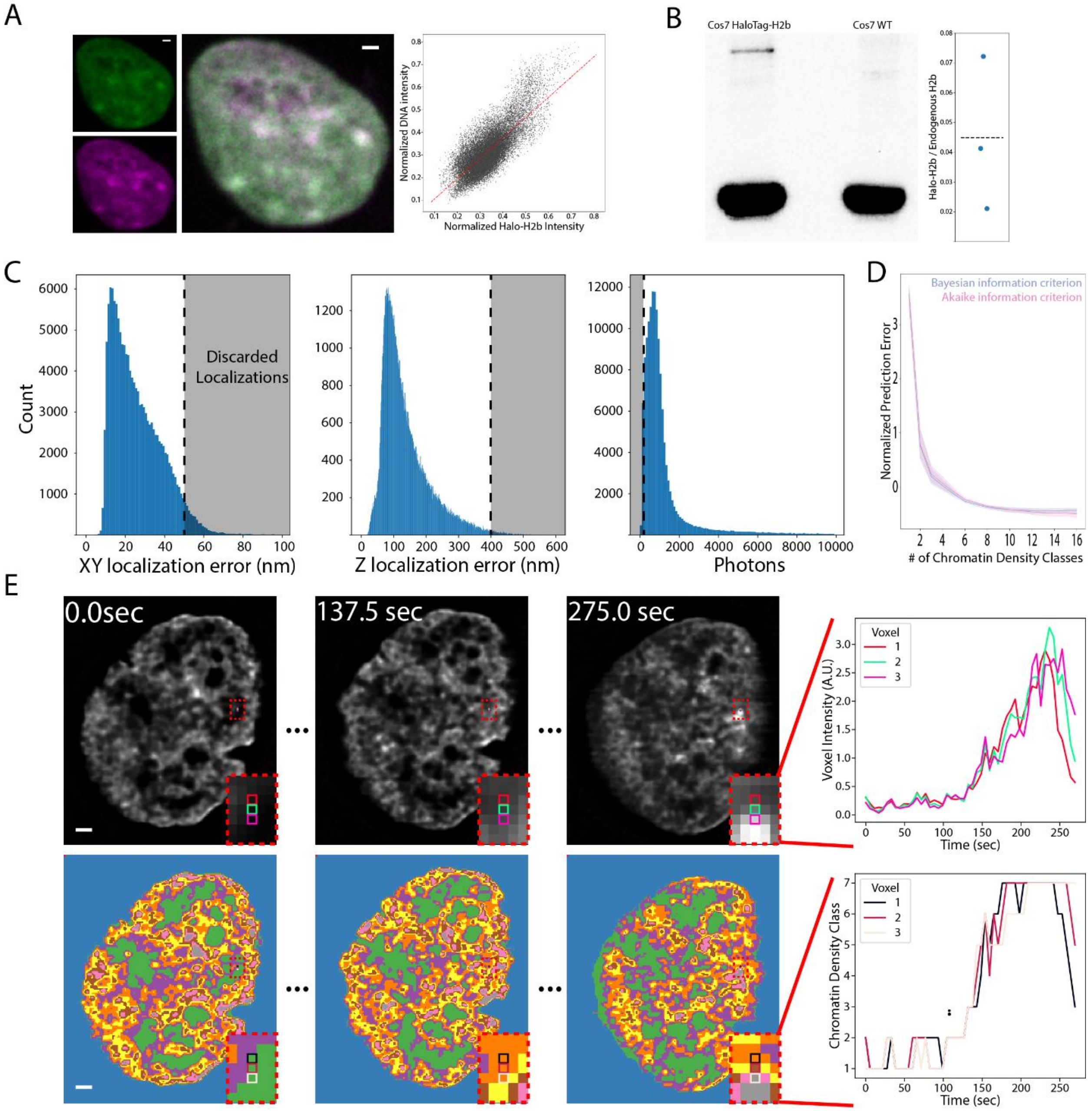

Supplementary Figure 1: (A) Confocal images of DNA (Green, Hoechst) and HaloTag-H2B (Magenta, HTL-JF647) in Cos7-Halo-H2B cells. The middle panel shows the merged image. The right panel shows the pixel-wise intensity correlation between the two. Scale bars = 1000 nm. The red line shows the linear fit of the data, with $R^2 = 0.77$. (B) Example western blot to quantify the ratio of HaloTag-H2B to endogenous H2B in Cos7-Halo-H2B cells and Cos7 wildtype cells. Quantification of the exogenous HaloTag-H2B band relative to endogenous H2B band. Blue dots represent one replicate and dashed line represents the mean of all replicates. (C) The distribution of nucleosomes' lateral (left) and axial (middle) localization error and the number of photons (right) for live-cell lattice light sheet imaging. Shaded areas are discarded by the post-processing localization filter. (D) Normalized Akaike information criterion (magenta) and Bayesian information

criterion (blue) for fitting of mixture of Gaussian model with increasing number of components to intensity histograms of deconvolved chromatin images. Values are z-score normalized. Solid lines represent the mean value and the shaded regions represent the 95% confidence interval. (E) Top panel: Representative deconvolved chromatin images taken at the beginning, halfway through and at end of imaging. Line plot indicating the fluorescence intensity (A.U. : arbitrary units) as a function of time (seconds) of set of voxels in a 3x1 line profile drawn in the deconvolved image. Bottom panel: Representative chromatin density classification images of top panel. Line plot indicating the chromatin density class as a function of time (seconds) for the same set of voxels in top panel. Scale bars = 1000 nm. Data in (A) are from a single representative cell. Data in (B) are from n = 3 independent biological replicates. Data in (C) are representative distributions from a single cell. Data in (D) are from n = 20 cells from 1 biological replicate. Data in (E) are from a single representative cell.

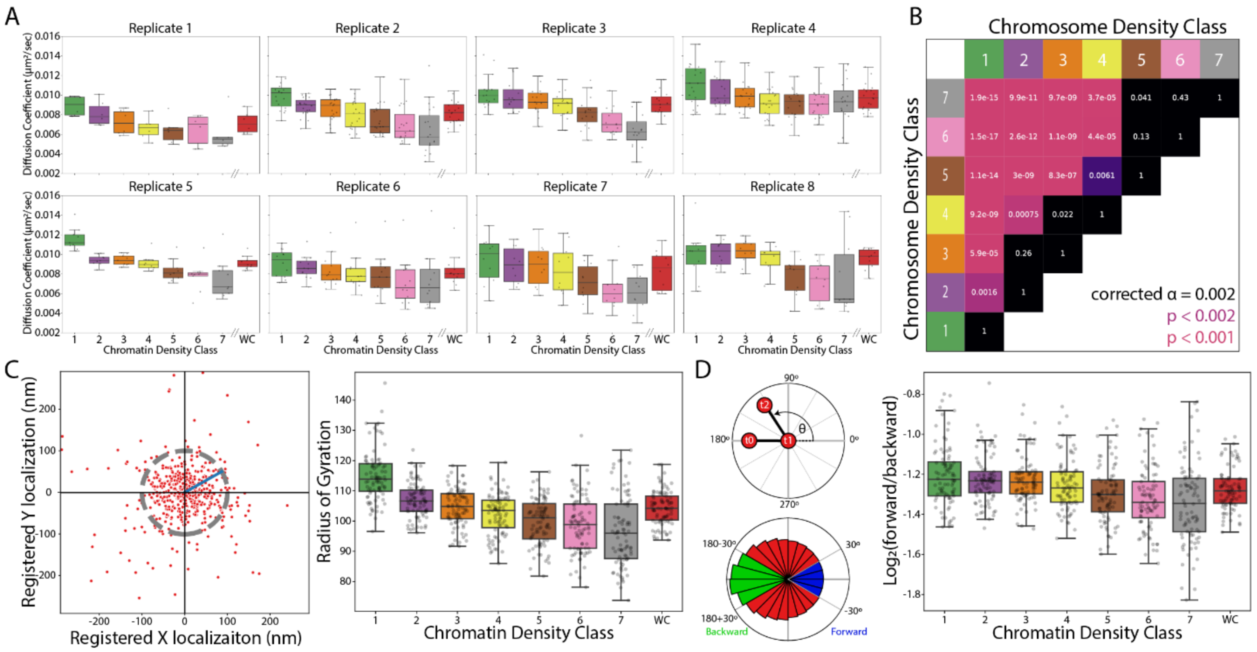

Supplementary Figure 2: (A) Box plot of the apparent diffusion coefficient of nucleosome motion in different chromatin classes across multiple experimental replicates. The plot follows that same convention as Figure 2B. WC indicates whole cell. (B) Pair-wise t-test of nucleosome diffusion coefficient between different chromatin classes. Corrected α corresponds to a p-value = 0.05 corrected for multiple hypotheses according to Bonferroni correction. (C) Left: Schematics illustrating the calculation of the radius of gyration. Red circles represent individual localizations, registered to the origin. The derived radius of gyration is depicted as a blue line, which in turn defines the gray circle centered around the origin. Right: box plot of radius of gyration in different chromatin density classes, plot follows the same convention as Figure 2B. (D) Left: schematics for calculating the anisotropy of nucleosome motion. θ is defined as the angle between two consecutive steps. For θ between -30 to 30 degrees, the motion is defined as forward; for θ between 150 to 210 degrees, the motion is defined as backward. Right: box plot of the ratio of fold change of forward/backward portion in different chromatin classes. Plot follows the same convention as (C). Data are from n = 88 cells across 8 independent biological replicates.

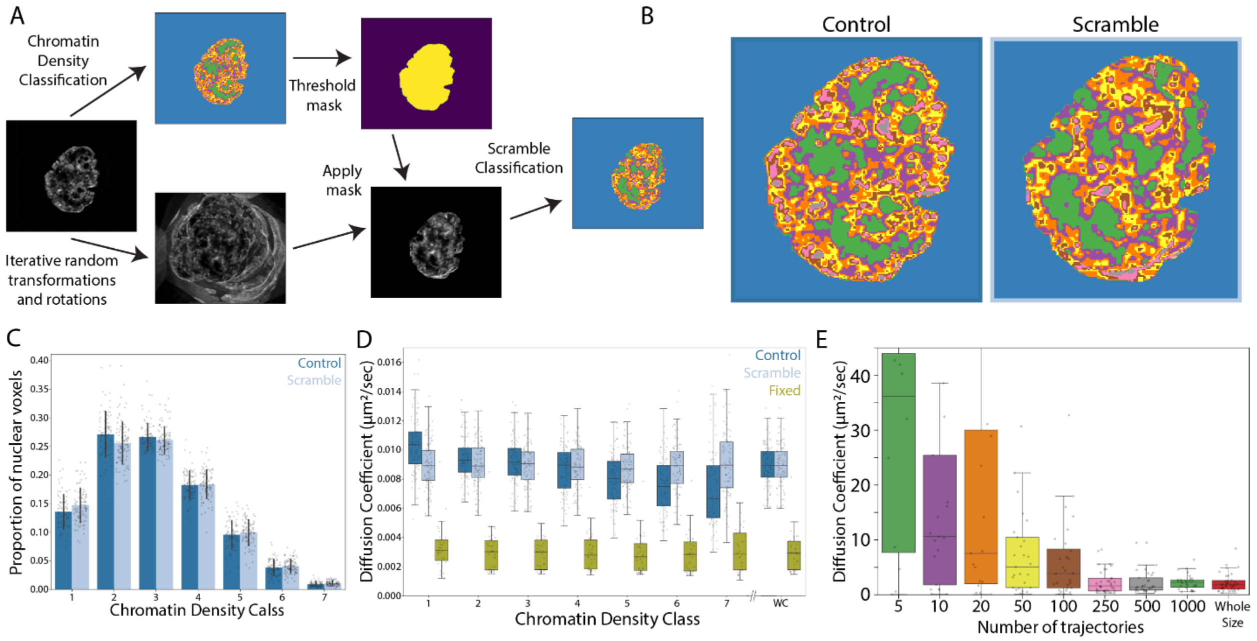

Supplementary Figure 3: (A) Schematic for generating scrambled chromatin classes. A scrambled, space filling image is generated through iteratively adding a transformed and rotated chromatin image. This randomly transformed image is masked, and chromatin density classification is performed. (B) Comparison between the real chromatin density classes (left) and the scrambled chromatin density classes (right). (C) Histogram of nuclear voxels associated with chromatin density classes for chromatin (dark blue) and scrambled (light blue) classes. Bar height indicates the mean proportion of voxels in a given class. The error bars indicate standard deviation. The dots represent measurement in a single cell. (D) Box plot of diffusion coefficient in control (dark blue, same as Figure 2B) and scrambled (light blue) classes. The yellow boxes show the diffusion coefficient of nucleosomes in chemically fixed cells. (E) Box plot of nucleosome diffusion coefficients' dependence on sample size. The x axis indicates the number of trajectories to which each sample was down sampled, and "whole size" indicates no down sampling. Data in (C) and (D) are from n = 88 cells across 8 independent biological replicates (Control), n = 88 cells across 8 independent biological replicates (Scramble), n = 34 cells across 3 independent biological replicates (Fixed). Data from (E) are from n = 37 cells across 4 independent biological replicates.

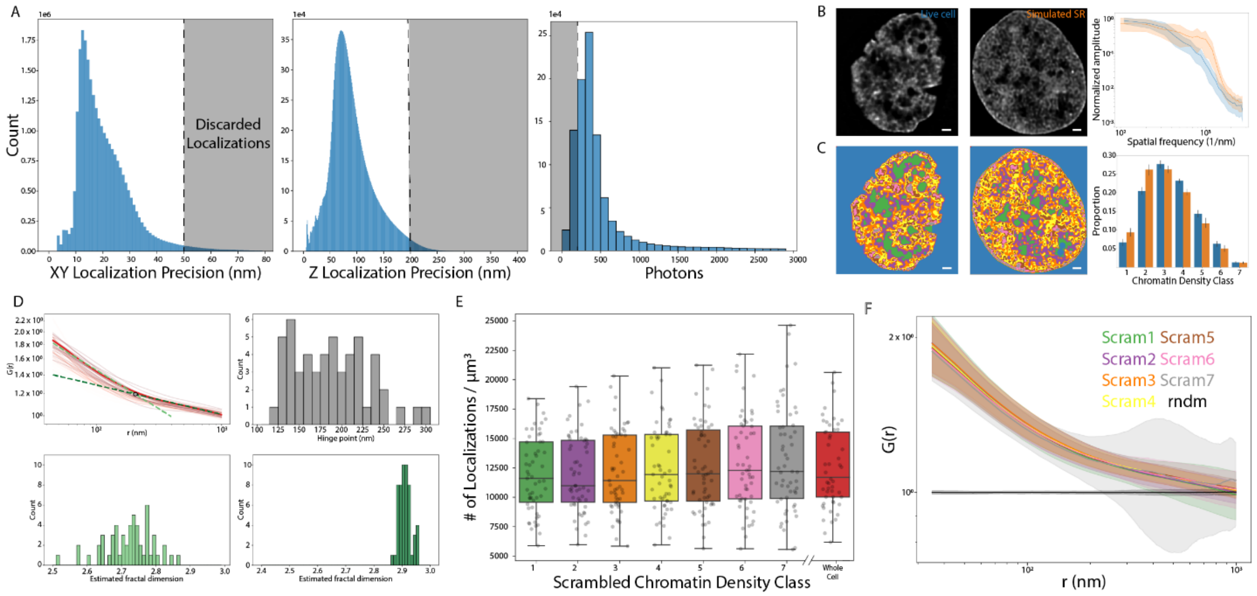

Supplementary Figure 4: (A) The histogram of nucleosomes' lateral (left) and axial (middle) localization error, and the number of photons (right) for fixed cell HiST single molecule localization microscopy. Shaded areas are discarded by the post-processing localization filter. (B) Comparison of image power spectrum between images taken with lattice light sheet microscopy and comparable images generated by convolving the super resolution single molecule localization microscopy dataset with a lattice light sheet microscopy point spread function. Left panel: representative image of deconvolved chromatin structure in live cell taken with lattice light sheet microscopy (same as Figure 1E); Middle pane: representative image generated using the single-molecule localizations (same as Figure 3B). Scale bars = 1000 nm; Right panel: power spectrum of the lattice light sheet microscopy (blue) and convolved single-molecule images (orange). Bar represents mean normalized amplitude and shaded region represents standard deviation. (C) The corresponding chromatin density class distribution in (B). (D) Estimated fractal dimension based on the pair correlation function (G(r)) calculated over the whole cell. Top left: representative G(r) of whole cells. The thick red indicates G(r) curve for an example cell. The opaque lines represent G(r) curves for other cells. Black circle indicates the hinge point separating the two fitting regimes. The light green line shows the power law fitting over the length scale smaller than the hinge point and the dark green line shows the power law fitting over the length scale larger than the hinge point. Top right: the histogram of hinge points. Bottom left: the histogram of the estimated fractal dimensions smaller than the hinge point. Bottom right: the histogram of the fractal dimensions larger than the hinge point. (E) Box plot of nucleosome localization density in different scrambled chromatin classes. The plot follows that same convention as Figure 2B. (F) Pair correlation function of the nucleosome organization for scrambled chromatin classes. The black curve is the G(r) for a random distribution. The plot follows that same convention as Figure 3L. Data in A are from a single representative cell from 1 biological replicate. Data in B are from n = 20 cells (LLSM) or n = 17 cells (HiST) across a 1 independent biological replicate. Data in (D)-(F) are from n = 54 cells across 3 independent biological replicates.

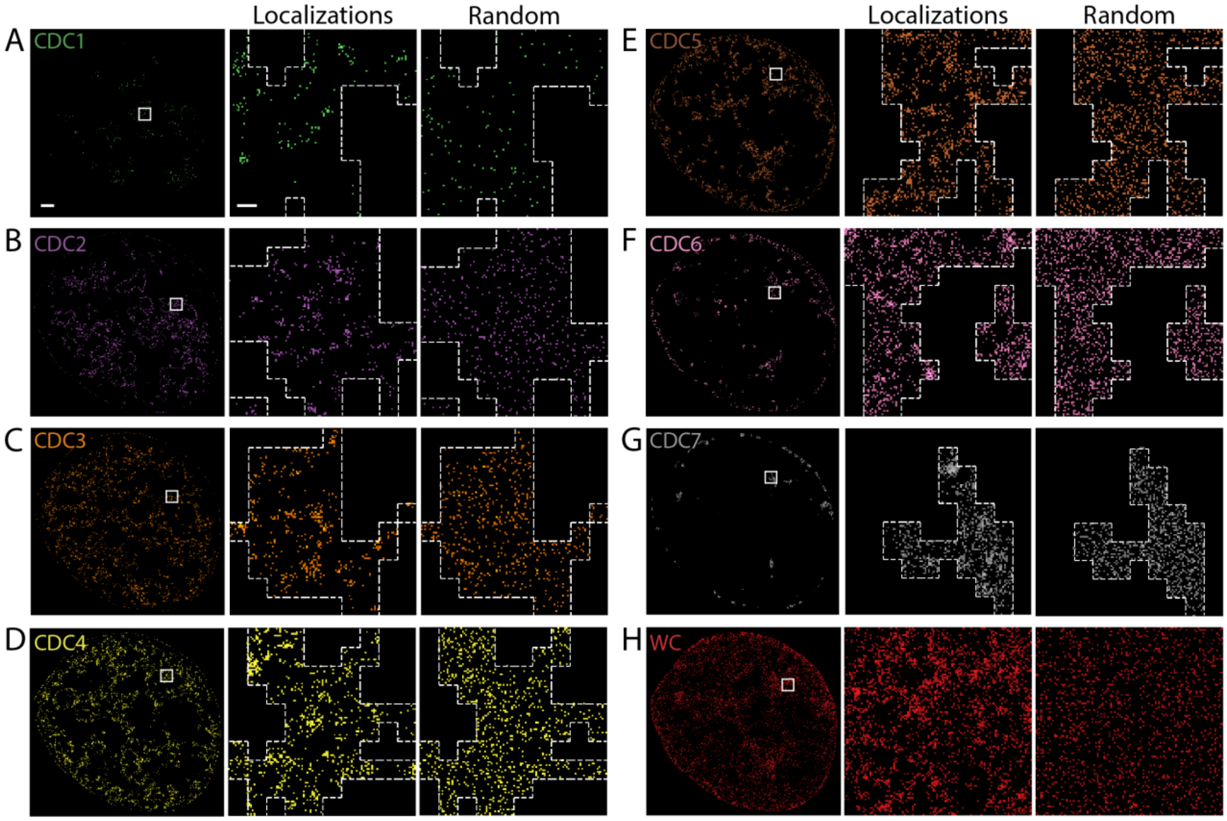

Supplementary Figure 5: Representative comparisons of the experimental and randomized nucleosome distribution in different chromatin density classes. (A) Left panel: nucleosome localization associated with chromatin density class 1. Middle panel: zoom-in of the white box in the left panel, the dashed line shows chromatin class 1 boundary. Right panel: random distribution of nucleosome localization with the same density. (B-G) Visual comparison between experimental and randomized nucleosome distribution for chromatin density class 2 to 7 respectively, organized similar as (A). (H) Visual comparison between experimental and randomized nucleosome distribution of the whole cell, organized similar as (A). Scale bar in (A) left panel = 1000 nm and applies to all left panels. Scale bar in (A) middle = 100 nm and applies to all middle and right panels.

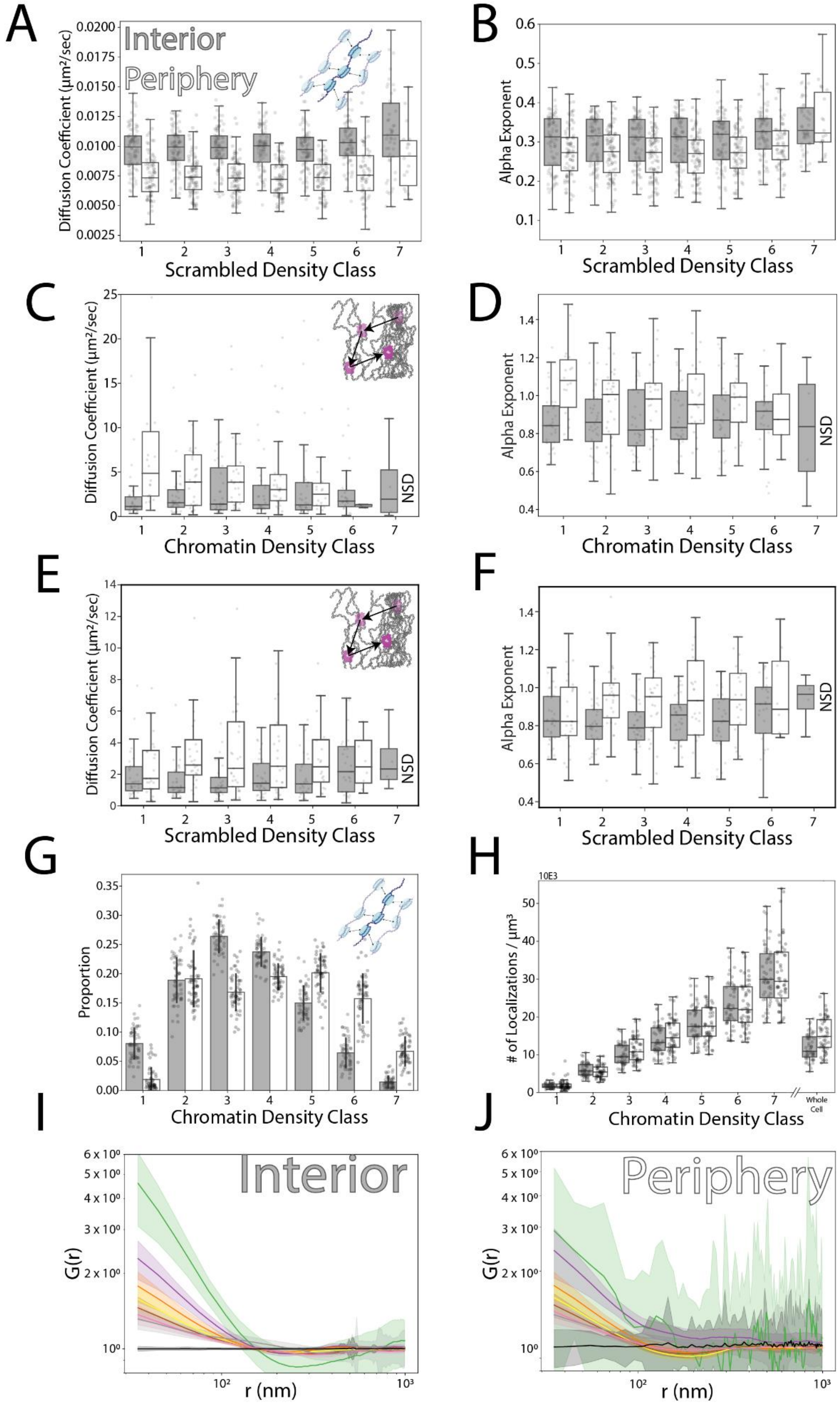
A
Interior
Periphery
Diffusion Coefficient (µm²/sec)
Scrambled Density Class
B
Alpha Exponent
Scrambled Density Class
C
Diffusion Coefficient (µm²/sec)
NSD
Chromatin Density Class
D
Alpha Exponent
NSD
Chromatin Density Class
E
Diffusion Coefficient (µm²/sec)
NSD
Scrambled Density Class
F
Alpha Exponent
NSD
Scrambled Density Class
G
Proportion
Chromatin Density Class
H
10E3
# of Localizations / µm³
Whole Cell
Chromatin Density Class
I
Interior
G(r)
r (nm)
J
Periphery
G(r)
r (nm)

Supplementary Figure 6: (A) Box plot of diffusion coefficient for nucleosomes in scrambled chromatin density classes in nuclear interior (Grey) and periphery (White). The plot follows the same color assignment and convention as Figure 4F. (B) Box plot of anomalous alpha exponent for nucleosomes in scrambled density chromatin classes. The plot follows that same color assignment and convention as (A). (C) Box plot of diffusion coefficient for HaloTag-NLS when every cell is subsampled to have 250 trajectories. The plot follows the same color assignment and convention as (A). (D) Box plot of anomalous exponent for HaloTag-NLS when every cell is subsampled to have 250 trajectories. The plot follows the same color assignment and convention as (A). (E) Box plot of diffusion coefficient for HaloTag-NLS in scrambled chromatin density classes. The plot follows the same color assignment and convention as (A). (F) Box plot of anomalous alpha exponent for HaloTag-NLS in scrambled density chromatin classes. The plot follows the same color assignment and convention as (A). “NSD” indicates not sufficient data, meaning the number of cells having more than 250 trajectories in that condition are less than three. (G) Histogram of voxels associated chromatin density classes for nuclear interior and nuclear periphery. The plot follows the same color assignments same as (A). The plot follows the same convention as Supplementary Figure 4C. (H) Box plot of localization density in different chromatin classes in the nuclear interior and periphery. The plot follows the same color assignment and convention as (A). (I) Pair correlation function of the nucleosome organization for different chromatin classes in the nuclear interior. The plot follows the same color assignment and plot convention as Figure 3J (J) Pair correlation function of the nucleosome organization for different chromatin classes in the nuclear periphery. The plot follows the same color assignment and plot convention as (I). Data in (A), (B), (E) and (F) are from n = 88 cells across 8 independent biological replicates. Data from (C) and (D) are from n = 37 cells across 4 independent biological replicates. Data from (G)-(J) are from n = 54 cells across 3 independent biological replicates.

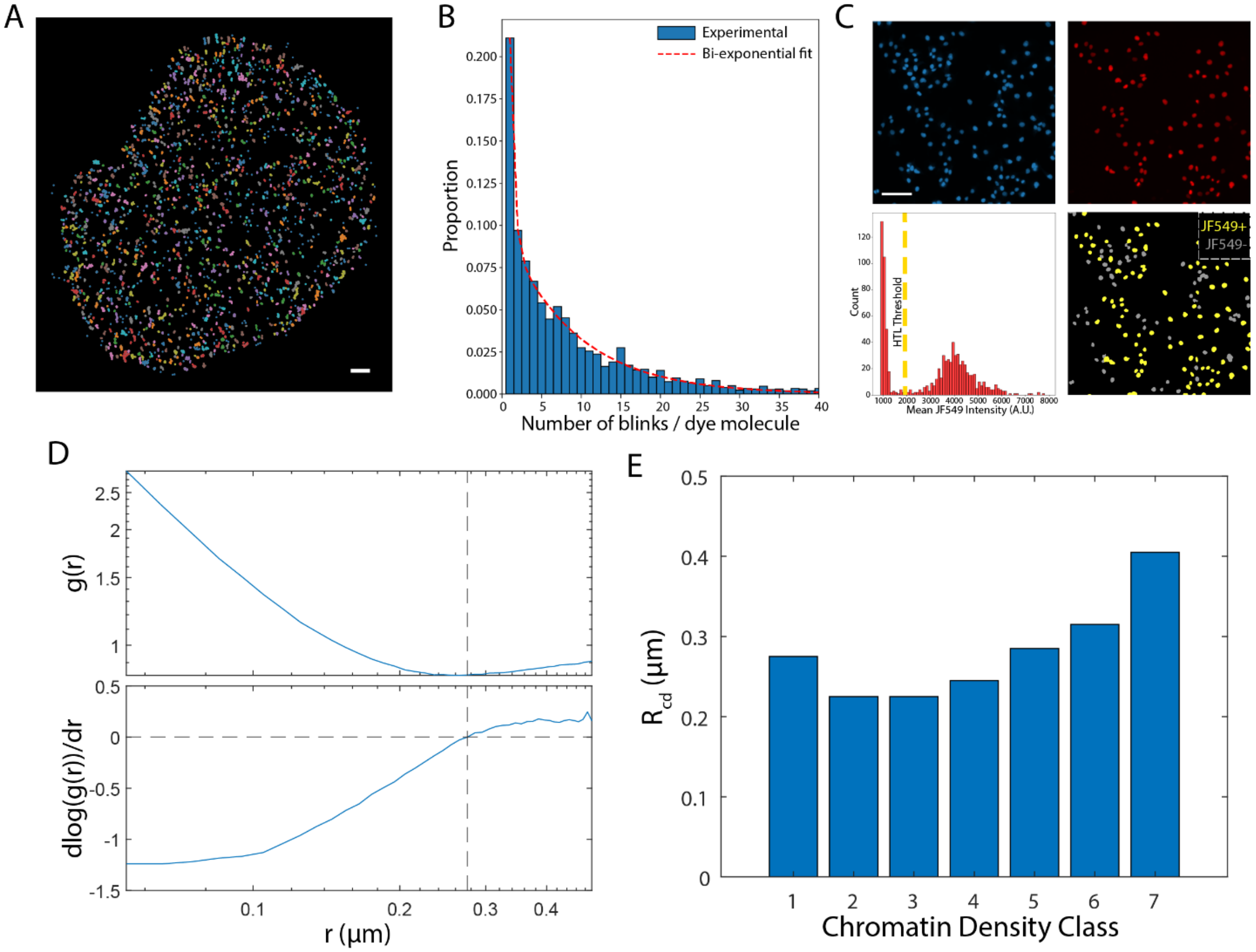

Supplementary Figure 7: (A) Characterization of the blinking properties for JF630B. Nucleosomes are sparsely labelled with the JF630B-HTL dye. All detected localizations are aggregated over 100,000 frames, and the density-based scan method is applied to determine clusters. Each cluster represents the blinking events for a single JF630b-HTL molecule. Scale bar = 1000 nm. (B) Histogram of the number of blinks in each JF630b labeled HaloTag-H2B molecule. The dashed line shows a bi-exponential fit to the histogram. (C) Epifluorescent images of Cos7-Halo-H2B cells labelled with Hoechst (blue, top left) and HTL-JF549 (red, top right). Scale bar = 100 µm. The bottom left panel shows the histogram of cell-averaged intensity of JF549, and the yellow dashed line shows the histogram below which cells are considered as not expressing HaloTag-H2B. The bottom right panel shows the segmented nuclei expressing HaloTag-H2B (yellow) and non-expressing (gray) cells. (D) Calculation of chromatin domain size used in the biophysical model. The top panel shows the G(r) of a representative cell in log-log scale, and the bottom plot shows the corresponding first derivative. The horizontal dashed line represents when the first derivative reaches zero, and the vertical dashed line is the corresponding radius which is extracted as an estimation of the chromatin domain size. (E) The distribution of chromatin domain size for different chromatin density classes. Data in A and B are from n = 13 cells across 2 independent biological replicates. Data in C are from n = 846 cells from 1 of 3 biological replicates. Data in E are from n = 54 cells across 3 independent biological replicates.

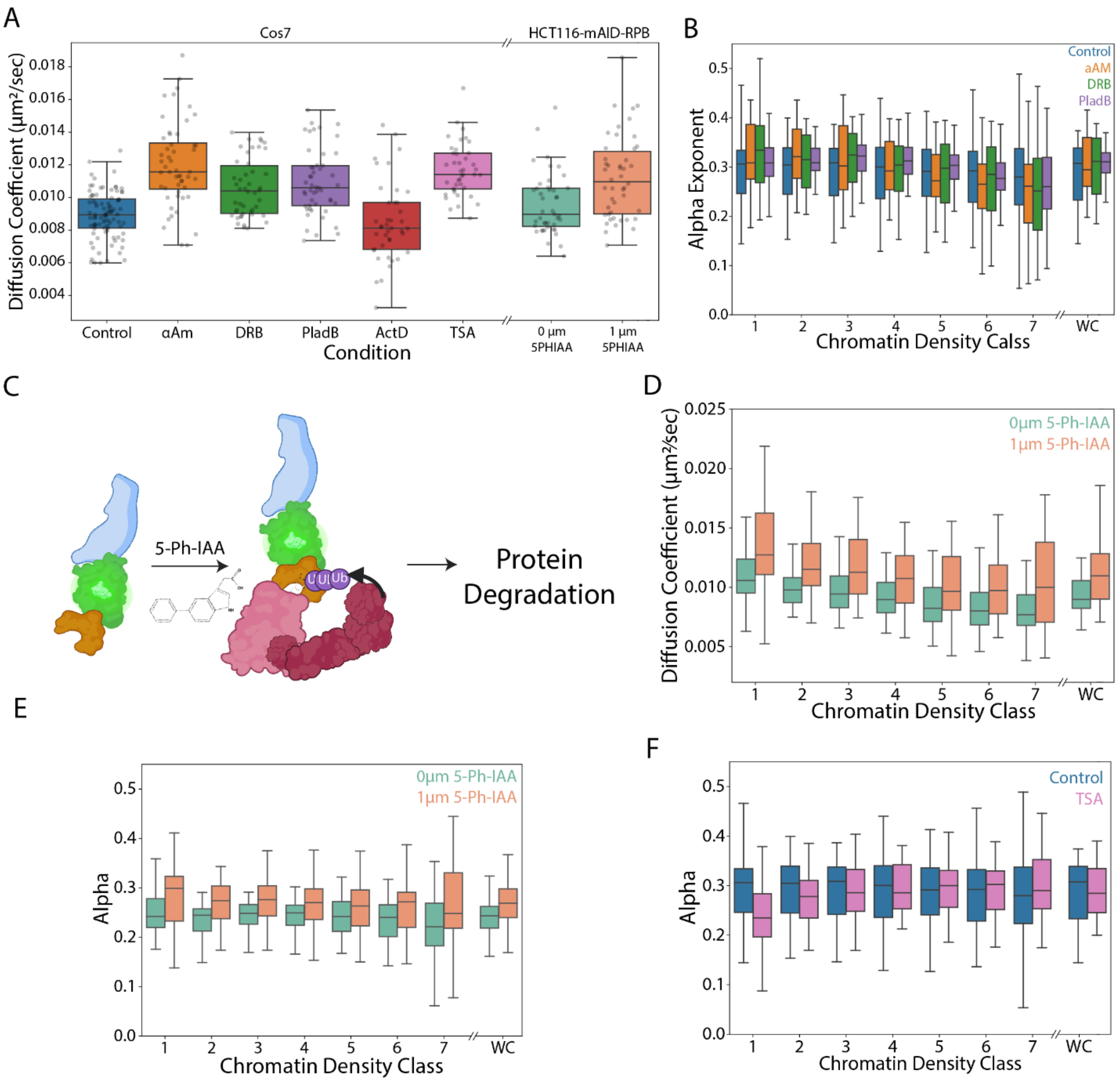

Supplementary Figure 8: (A) Box plot of whole cell averaged nucleosome diffusion coefficient for different pharmacological perturbations. The plot follows that same convention as Figure 2B. (B) Box plot of MSD anomalous alpha exponent for different chromatin classes under perturbations that inhibit gene transcription. The plot follows the same convention as Figure 6B. (C) A schematic of conditional knock down of the RNA polymerase II major subunit. (D) Box plot of diffusion coefficients of nucleosomes in different chromatin density classes in HCT116-mClover-mAID-RPB+Halo-H2B cells under control (green) and after conditional knockdown of RNA Polymerase major subunit with 1 µm 5-Ph-I-AA (coral). This plot follows the same convention as Figure 6B (E) Box plot of MSD exponent of nucleosomes in different chromatin density classes in HCT116 under control after conditional knock down of RNA polymerase major subunit. This plot follows the same color assignment and convention as (D) (F) Box plot of MSD exponent in different chromatin density classes under control (blue) and TSA (pink). The plot follows the same convention as (D). Data from (A), (B) and (F) are from n = 88 cells across 8 replicates (control), n = 52 cells across 3 replicates (α-amanitin), n = 46 cells across 3 replicates (DRB), n = 49 cells across 3 replicates (PladB), n = 41 cells across 3 replicates (ActD) and n = 60 cells across 3 independent replicates (TSA). Data from (D) and (E) are from n = 43 cells (0 µM 5PH-I-AA) and n = 46 cells (1 µm 5PH-I-AA) across 3 independent replicates.

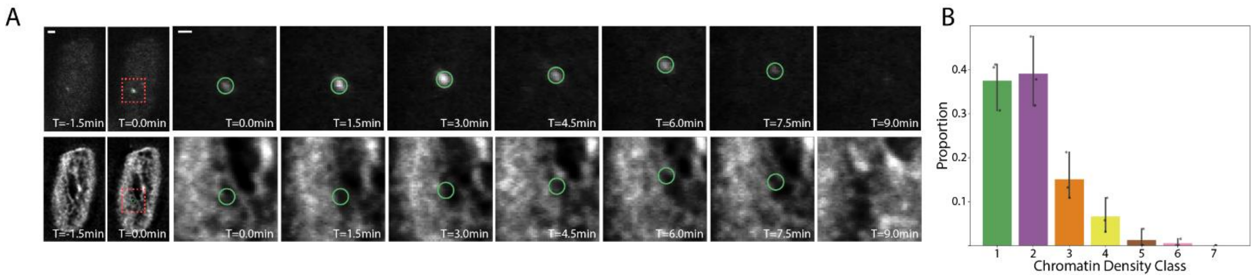

Supplementary Figure 9: (A) A representative example of TFF1 transcriptional burst in MCF7 cells. Top: GFP tagged MS2 coat protein aggregating at TFF1-MS2 transcriptional burst. Bottom: the corresponding chromatin image. Whole cell scale bar = 1000nm. Inset scale bar = 500nm (B) Histogram of the chromatin density class in which a TFF1 transcriptional burst initiates. The height of each bar represents the mean across replicates. The error line represents the standard deviation across replicates. The dots represent the mean for a single replicate. Data is from n = 105 cells over 3 independent replicates.

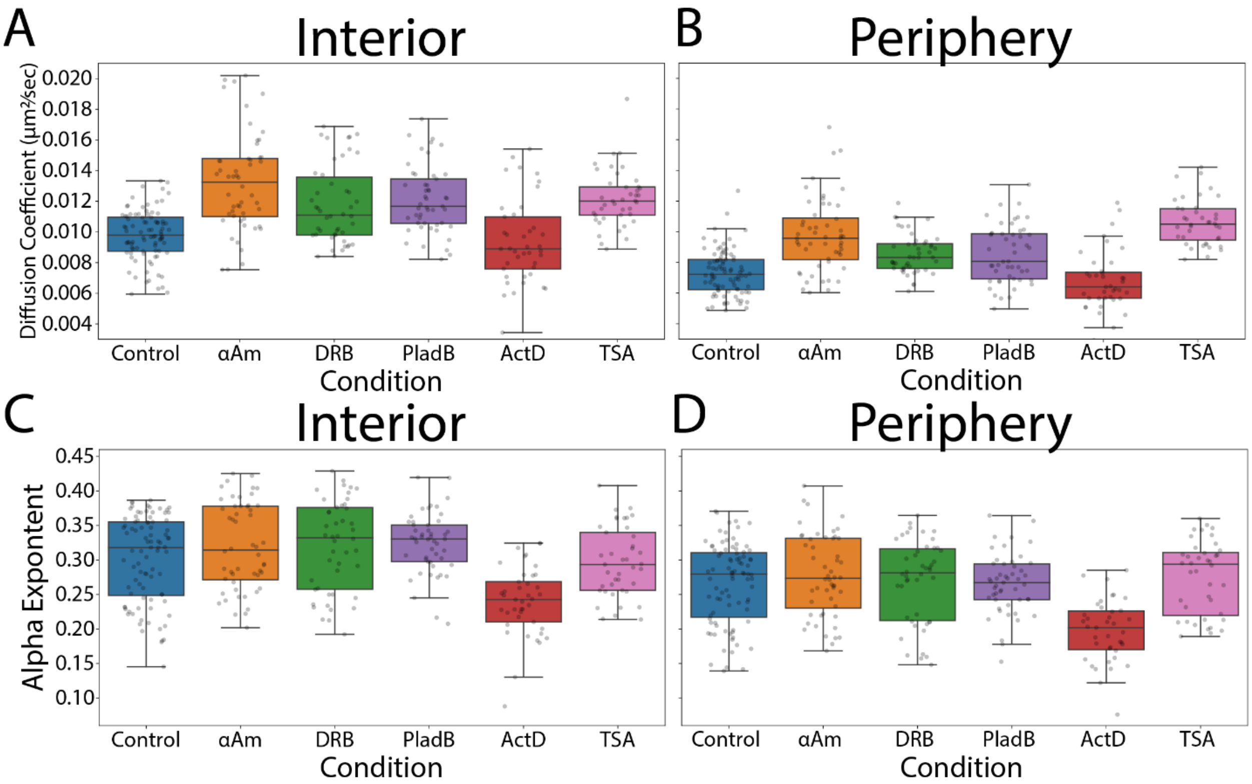

Supplementary Figure 10: (A) Box plot of extracted nucleosome diffusion coefficient in the nuclear interior under control (blue), α-amanitin (orange), DRB (green), PladB (purple), ActD (red) and TSA (Pink). (B) Box plot of extracted nucleosome diffusion coefficient in the nuclear periphery. The plot color assignment and convention follow the same as (A). (C) Box plot of extracted anomalous alpha exponent in the nuclear interior. The plot follows the same color assignment and convention as (A). (D) Box plot of extracted anomalous alpha exponent in the nuclear periphery. The plot follows the same color assignment and convention as (A). Data from A and B are from n = 88 cells across 8 replicates (control), n = 52 cells across 3 replicates (α-amanitin), n = 46 cells across 3 replicates (DRB), n = 49 cells across 3 replicates (PladB), n = 41 cells across 3 replicates (ActD) and n = 60 (TSA) cells across 3 independent replicates.

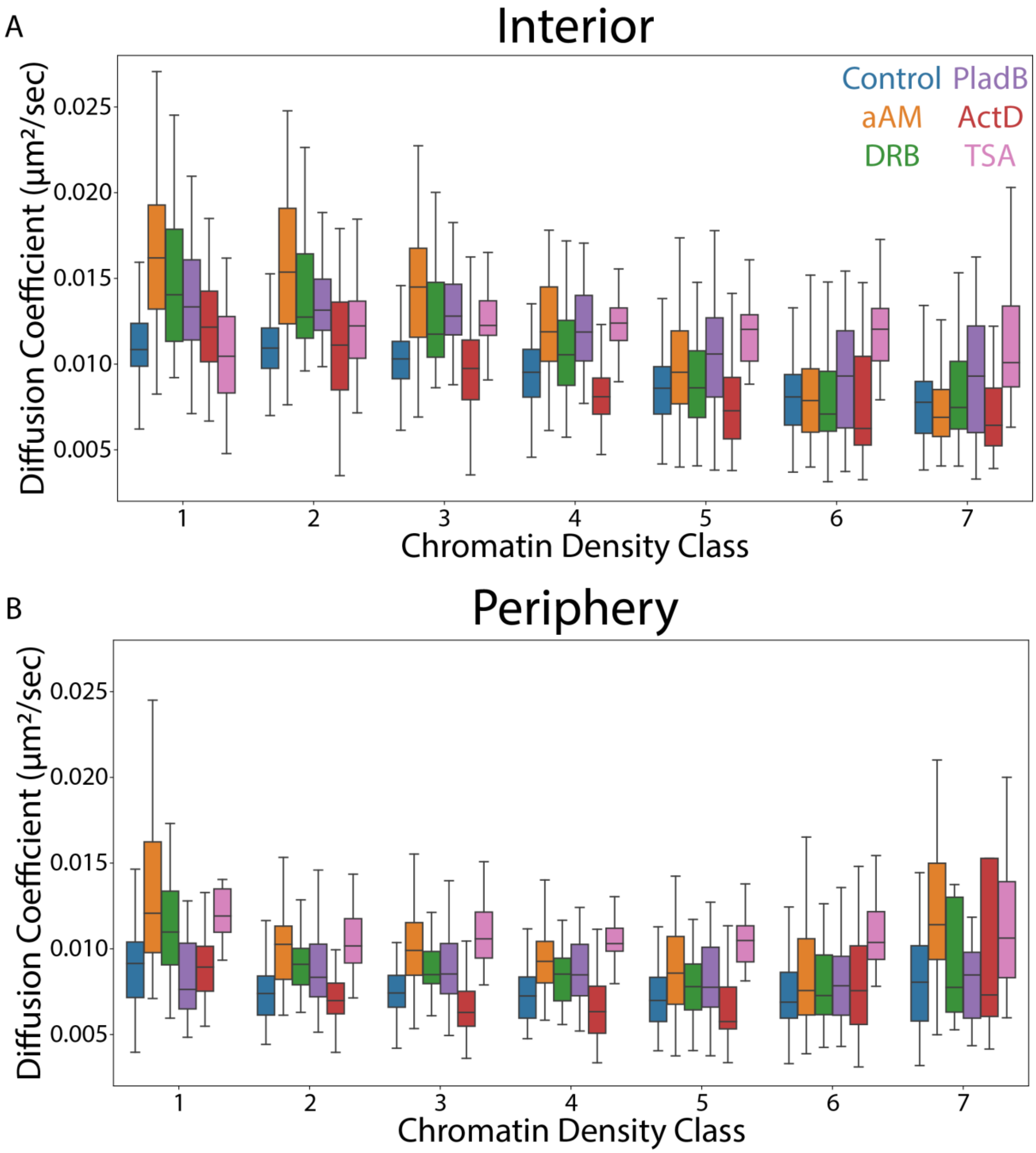

Supplementary Figure 11: (A) Box plot of extracted nucleosome diffusion coefficient in different chromatin density classes in the nuclear interior under control (blue), α-amanitin (orange), DRB (green) PladB (purple), ActD (red) and TSA (Pink). (B) Box plot of extracted nucleosome diffusion coefficient in different chromatin density classes in the nuclear periphery. The plot follows the same color assignment and convention same as (A). Data from A and B are from n = 88 cells across 8 replicates (control), n = 52 cells across 3 replicates (α-amanitin), n = 46 cells across 3 replicates (DRB), n = 49 cells across 3 replicates (PladB), n = 41 cells across 3 replicates (ActD) and n = 60 (TSA) cells across 3 independent replicates.

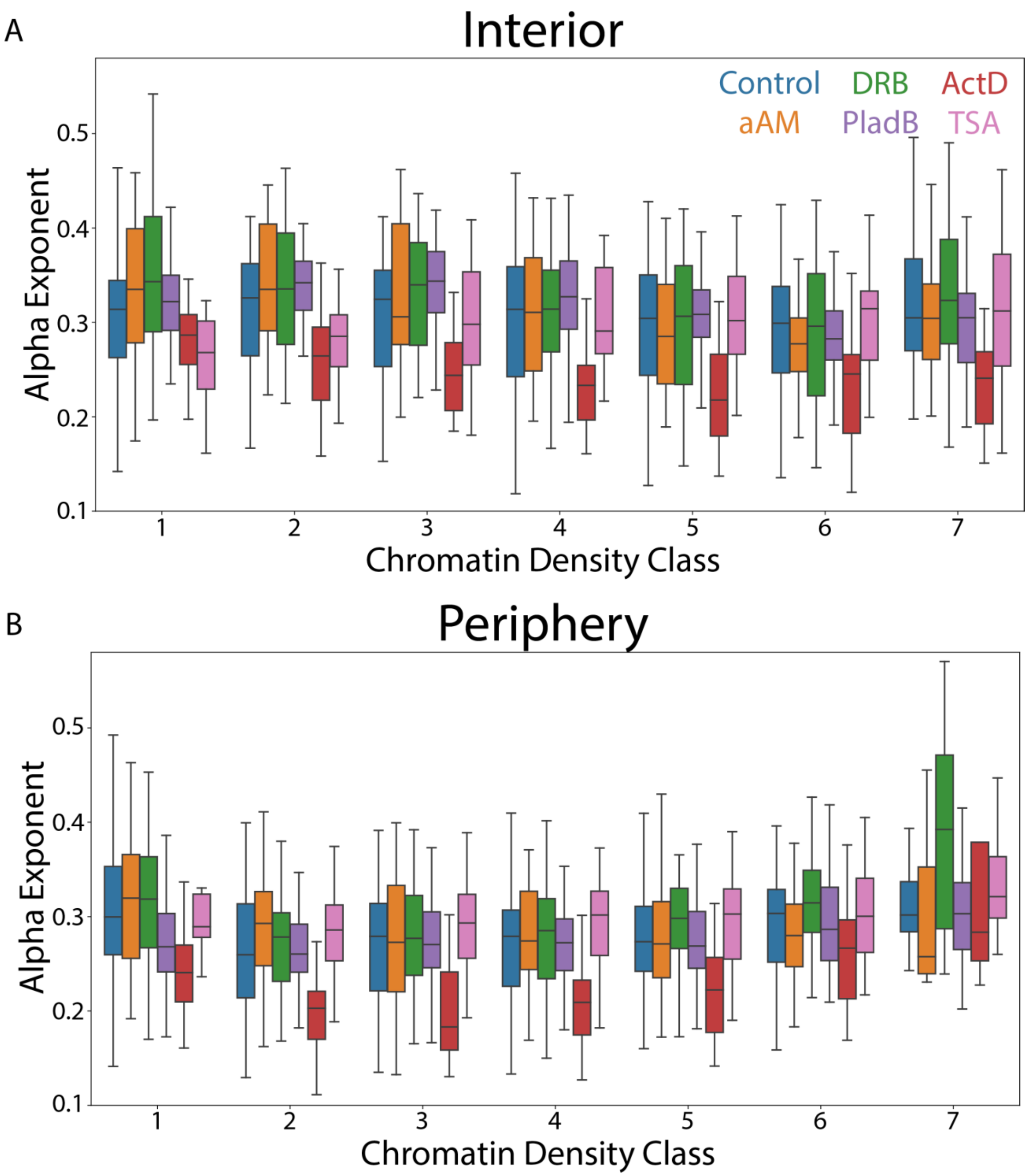

Supplementary Figure 12: (A) Box plot of the extracted nucleosome anomalous alpha exponent in different chromatin density classes in the nuclear interior. The plot follows the same color assignment and plot convention as Supplementary Figure 11A. (B) Box plot of the extracted nucleosome anomalous alpha exponent in different chromatin density classes in the nuclear periphery. The plot follows the same color assignment and convention as (A). Data from A and B are from n = 88 cells across 8 replicates (control), n = 52 cells across 3 replicates (α-amanitin), n = 46 cells across 3 replicates (DRB), n = 49 cells across 3 replicates (PladB), n = 41 cells across 3 replicates (ActD) and n = 60 (TSA) cells across 3 independent replicates.

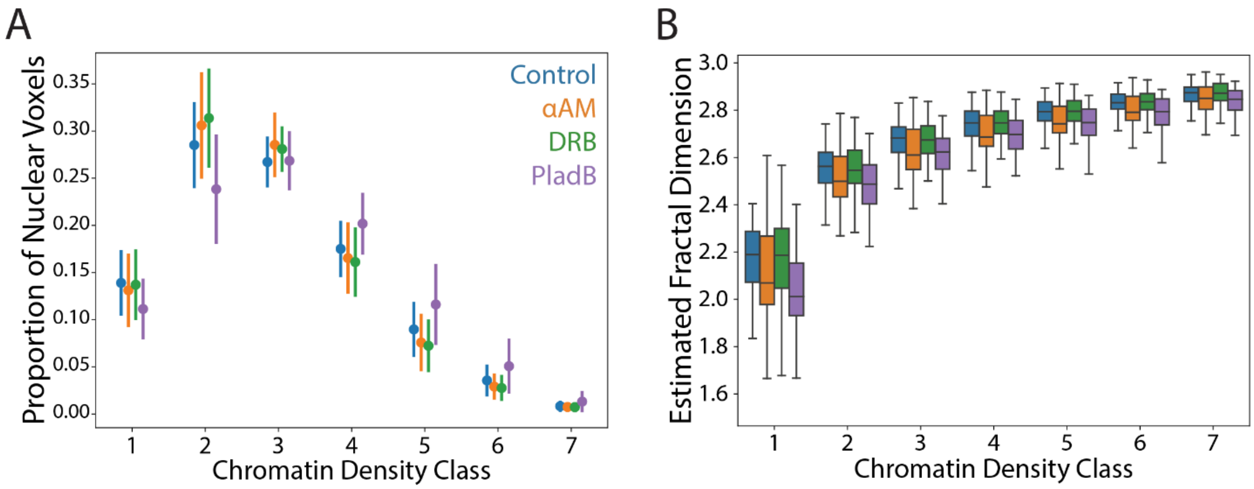

Supplementary Figure 13: (A) Distribution of nuclear voxels in different chromatin density classes under control (blue), α-amanitin (orange), DRB (green) and PladB (purple). The plot follows the same convention as Figure 6G. (B) Box plot of the estimated fractal dimension in different chromatin density classes under control (blue), α-amanitin (orange), DRB (green) and PladB (purple). This plot color assignments follow same as (A) The plot follows the same convention as Figure 6B. Data from (A) are from n = 88 cells (control), n = 52 cells (αAM), n = 46 cells (DRB), and n = 49 cells PladB across 3 independent replicates. Data from B are from n = 54 cells (control), n = 46 cells (αAM), n = 68 cells (DRB), and n = 61 cells (PladB) across 3 independent replicates.

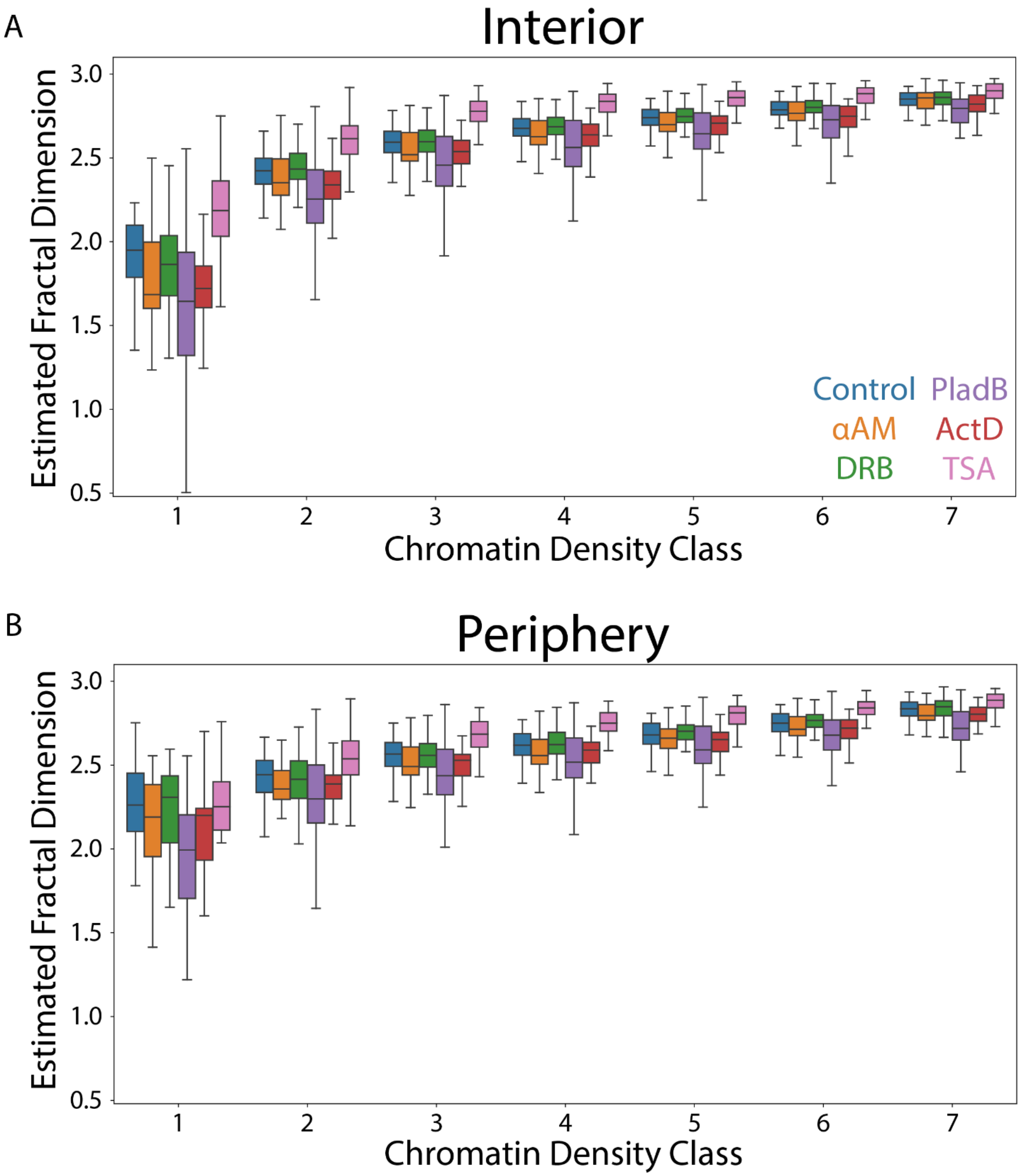

Supplementary Figure 14: (A) Estimated fractal dimension in chromatin density classes in the nuclear interior after drug perturbation. The plot follows the same color assignment and plot convention as Supplementary Figure 11A. (B) Estimated fractal dimension in chromatin density classes in the nuclear periphery after drug perturbation. Data are from n = 54 cells (control), n = 46 cells (αAM), n = 68 cells (DRB), and n = 61 cells (PladB) n = 54 cells (ActD), and n = 60 cells (TSA) across independent biological replicates. Box plots in (A) and (B) include only points from line fits with $r^2 > 0.95$.

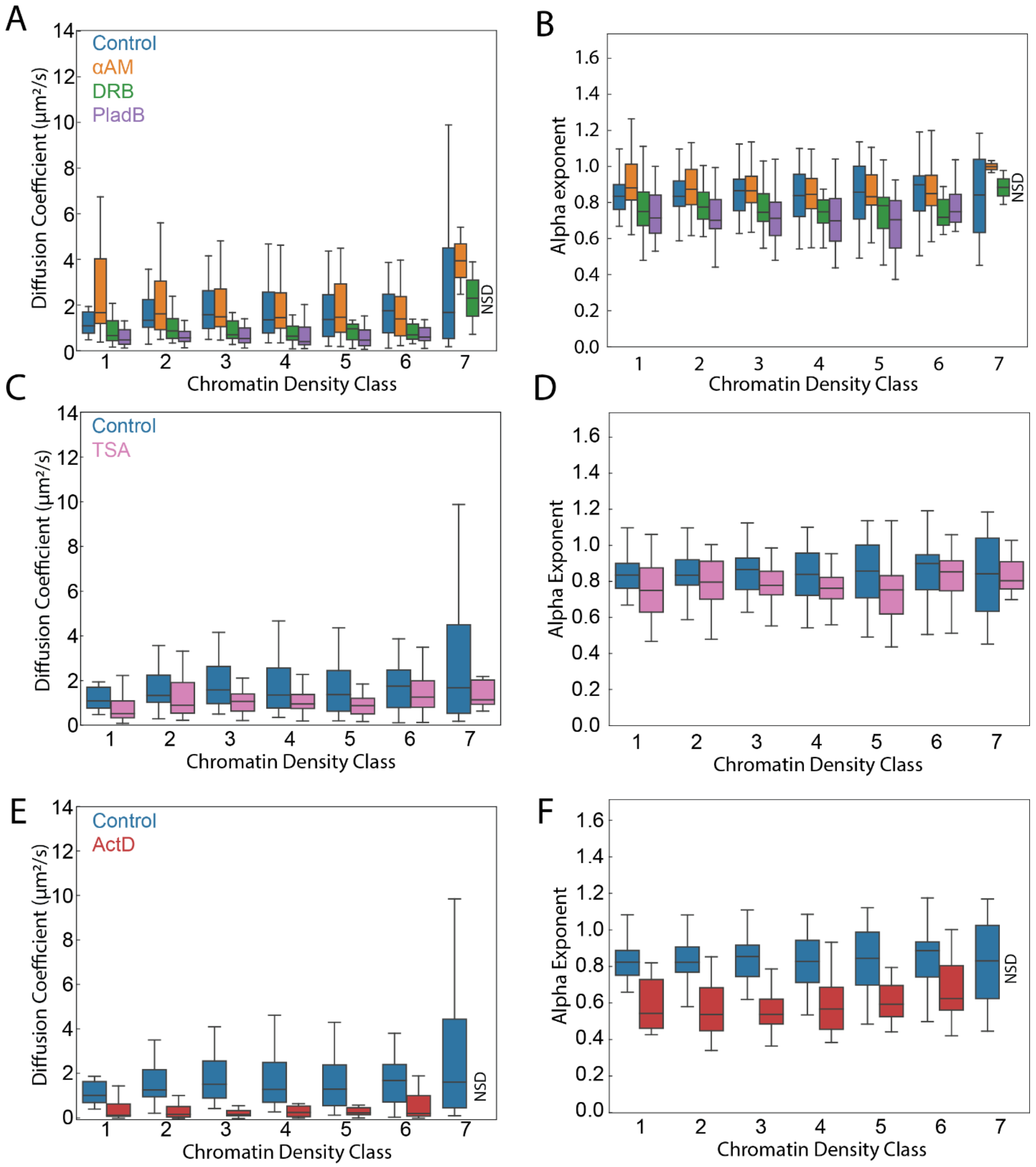

Supplementary Figure 15: (A, B) Box plots of the apparent diffusion coefficient (C) and the anomalous alpha exponent (D) of HaloTag-NLS in different chromatin density classes under control (blue), α-amanitin (orange), DRB (green) and PladB (purple). The plot follows the same convention Figure 6B. (C-D) Box plots of the apparent diffusion coefficient and anomalous alpha exponent of HaloTag-NLS in different chromatin density classes under control (blue) and TSA (pink). The plot follows the same convention as Figure 6F. (E-F) Box plots of the apparent diffusion coefficient and anomalous alpha exponent of HaloTag-NLS in different chromatin density classes under control (blue) and ActD (red). Theses plots follow the same convention as Figure 6 C,D. Data are from n = 37 cells (control), n = 36 cells (α-amanitin), n = 23 cells (DRB), n = 47 cells (PladB), n = 29 cells (ActD) and n = 42 cells (TSA). Data for control are across four independent replicates, and all other conditions are across three independent replicates. Box plots in (A)-(F) include only points from line fits to data that include at least 250 trajectories, and "NSD" indicates not sufficient data.

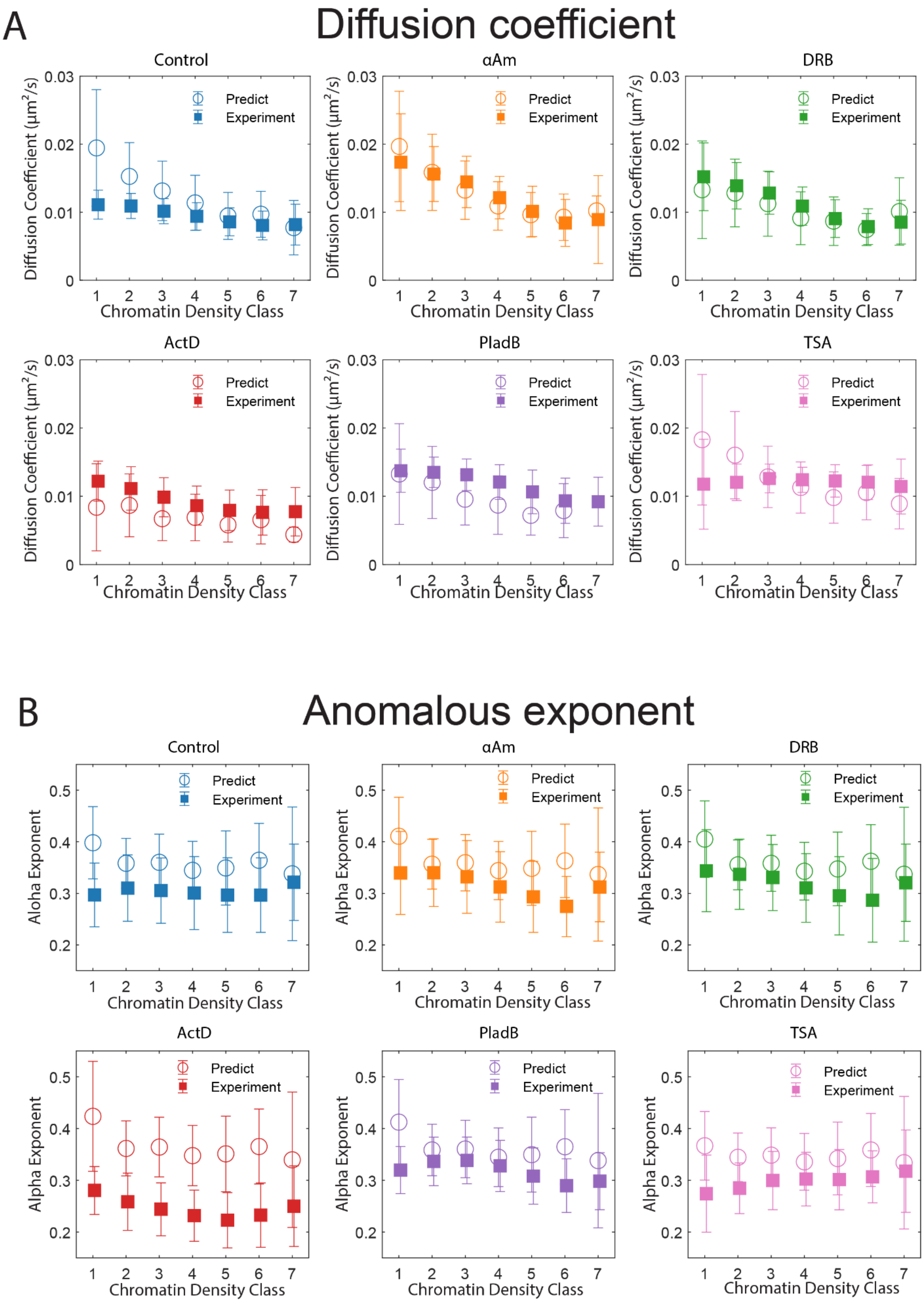
A
Diffusion coefficient
Control
αAm
DRB
ActD
PladB
TSA
Predict
Experiment
Diffusion Coefficient (μm²/s)
0.03
0.02
0.01
0
1 2 3 4 5 6 7
Chromatin Density Class
B
Anomalous exponent
Control
αAm
DRB
ActD
PladB
TSA
Predict
Experiment
Aloha Exponent
Alpha Exponent
0.5
0.4
0.3
0.2
1 2 3 4 5 6 7
Chromatin Density Class

Supplementary Figure 16: (A) Comparison of diffusion coefficients between model (open circles) and experiments (closed squares) under control (blue), α-amanitin (orange), DRB (green), ActD (red), PladB (purple), and TSA treatment (pink). Plots follow the same convention as Figure 7A. (B) Comparison of the anomalous alpha exponent. The plots follow same conventions as (A)
