# Supplementary material for: Correlative single molecule lattice light sheet imaging reveals the dynamic relationship between nucleosomes and the local chromatin environment
Source: Nat Commun. 2024 May 16;15:4178. doi: 10.1038/s41467-024-48562-0 (PMC11099156; doi:10.1038/s41467-024-48562-0)
Supplement: Supplementary file 3 — Description of Additional Supplementary Files [file 41467_2024_48562_MOESM3_ESM.pdf]

Title: Supplementary Movie 1:
Description: 3D lattice light sheet microscopy and classification of chromatin density in live cells.

Title: Supplementary Movie 2:
Description: Correlative single molecule tracking and lattice light sheet microscopy of nucleosomes and chromatin.

Title: Supplementary Movie 3:
Description: 3D highly inclined and swept tile imaging and classification of nucleosome organization.

Title: Supplementary Movie 4:
Description: Correlative single molecule tracking and lattice light sheet microscopy of free diffusing Halotag-NLS and chromatin.

Title: Supplementary Movie 5:
Description: Multicolor lattice light sheet microscopy of transcriptional bursts and chromatin.

Title: Supplementary Movie 6
Description: Processing steps chromatin images from lattice light sheet microscope.
