# Supplementary material for: Correlative single molecule lattice light sheet imaging reveals the dynamic relationship between nucleosomes and the local chromatin environment
Source: Nat Commun. 2024 May 16;15:4178. doi: 10.1038/s41467-024-48562-0 (PMC11099156; doi:10.1038/s41467-024-48562-0)
Supplement: Supplementary file 10 — Reporting Summary [file 41467_2024_48562_MOESM10_ESM.pdf]

nature portfolio

Corresponding author(s): Wesley R. Legant

Last updated by author(s): Apr 11, 2024

Reporting Summary

Nature Portfolio wishes to improve the reproducibility of the work that we publish. This form provides structure for consistency and transparency in reporting. For further information on Nature Portfolio policies, see our Editorial Policies and the Editorial Policy Checklist.

Statistics

For all statistical analyses, confirm that the following items are present in the figure legend, table legend, main text, or Methods section.

n/a	Confirmed	
☐	☒	The exact sample size (*n*) for each experimental group/condition, given as a discrete number and unit of measurement
☐	☒	A statement on whether measurements were taken from distinct samples or whether the same sample was measured repeatedly
☐	☒	The statistical test(s) used AND whether they are one- or two-sided<br>*Only common tests should be described solely by name; describe more complex techniques in the Methods section.*
☒	☐	A description of all covariates tested
☐	☒	A description of any assumptions or corrections, such as tests of normality and adjustment for multiple comparisons
☐	☒	A full description of the statistical parameters including central tendency (e.g. means) or other basic estimates (e.g. regression coefficient) AND variation (e.g. standard deviation) or associated estimates of uncertainty (e.g. confidence intervals)
☐	☒	For null hypothesis testing, the test statistic (e.g. *F*, *t*, *r*) with confidence intervals, effect sizes, degrees of freedom and *P* value noted<br>*Give P values as exact values whenever suitable.*
☒	☐	For Bayesian analysis, information on the choice of priors and Markov chain Monte Carlo settings
☒	☐	For hierarchical and complex designs, identification of the appropriate level for tests and full reporting of outcomes
☐	☒	Estimates of effect sizes (e.g. Cohen's *d*, Pearson's *r*), indicating how they were calculated

Our web collection on statistics for biologists contains articles on many of the points above.

Software and code

Policy information about availability of computer code

Data collection	For all Image and statistical analysis, we used established algorithms that were written in Matlab (version 2021b, 2022a, 2022b or 2023a), Python (version 3.7, 3.8 or 3.9) or R (Versions 4.1.2 or 4.2.2) as described in Methods.
Data analysis	For all Image and statistical analysis, we used established algorithms that were written in Matlab (version 2021b, 2022a, 2022b or 2023a), Python (version 3.7, 3.8 or 3.9) or R (Versions 4.1.2 or 4.2.2) as described in Methods.

For manuscripts utilizing custom algorithms or software that are central to the research but not yet described in published literature, software must be made available to editors and reviewers. We strongly encourage code deposition in a community repository (e.g. GitHub). See the Nature Portfolio guidelines for submitting code & software for further information.

Data

Policy information about availability of data

All manuscripts must include a data availability statement. This statement should provide the following information, where applicable:

- Accession codes, unique identifiers, or web links for publicly available datasets
- A description of any restrictions on data availability
- For clinical datasets or third party data, please ensure that the statement adheres to our policy

Due to the inordinate size of the image data (~50TB), it is not currently feasible to deposit this into a central repository; however, all datasets underlying the results in this manuscript are available from the corresponding author upon request. To the extent possible, the authors will try to meet all requests for data sharing within 2 weeks from the original request.

Research involving human participants, their data, or biological material

Policy information about studies with human participants or human data. See also policy information about sex, gender (identity/presentation), and sexual orientation and race, ethnicity and racism.

Reporting on sex and gender	N/A
Reporting on race, ethnicity, or other socially relevant groupings	N/A
Population characteristics	N/A
Recruitment	N/A
Ethics oversight	N/A

Note that full information on the approval of the study protocol must also be provided in the manuscript.

Field-specific reporting

Please select the one below that is the best fit for your research. If you are not sure, read the appropriate sections before making your selection.

☒ Life sciences ☐ Behavioural & social sciences ☐ Ecological, evolutionary & environmental sciences

For a reference copy of the document with all sections, see nature.com/documents/nr-reporting-summary-flat.pdf

Life sciences study design

All studies must disclose on these points even when the disclosure is negative.

Sample size	Sample sizes were not predetermined through statistical analysis. Instead, we based our sample size decisions on standard practices within the field, aiming for a minimum of three independent biological replicates for each experimental condition. This aligns with common benchmarks in cell biology that recognize three replicates as the minimum necessary to identify consistent patterns and reduce the impact of biological variability. Within each biological replicate, we aimed for observations in at least 12 independent cells. This would ensure that we would have sufficient data to run parametric statistical tests.
Data exclusions	Specific instances in which data points were excluded are noted with appropriate explanation and justification in the manuscript. In general, data were excluded when insufficient data remained after processing to draw reliable conclusions. All decisions to exclude data were made prior to statistical testing to prevent bias.
Replication	All datasets have at least three independent trials. Each trial contains at least 12 different cells. The specific number of cells used for each condition are reported in figure legends and in Methods.
Randomization	For all experiments and conditions, imaged cells were randomly selected to ensure unbiased distribution across control and treatment groups involving pharmacological perturbations. Covariate influences, such as cell size and shape, are inherently controlled by our random selection.
Blinding	Blinding was not necessary because the selection of cells for imaging was random, and all subsequent data processing and analysis were uniformly applied. This standardization minimizes subjective bias, as the same image processing pipeline and analytical procedures were applied to all samples, ensuring consistency across all experimental conditions.

Reporting for specific materials, systems and methods

We require information from authors about some types of materials, experimental systems and methods used in many studies. Here, indicate whether each material, system or method listed is relevant to your study. If you are not sure if a list item applies to your research, read the appropriate section before selecting a response.

Materials & experimental systems

n/a	Involved in the study	
☐	☒	Antibodies
☐	☒	Eukaryotic cell lines
☒	☐	Palaeontology and archaeology
☒	☐	Animals and other organisms
☒	☐	Clinical data
☒	☐	Dual use research of concern
☒	☐	Plants

Methods

n/a	Involved in the study	
☒	☐	ChIP-seq
☒	☐	Flow cytometry
☒	☐	MRI-based neuroimaging

Antibodies

Antibodies used	RRID:AB_302612 ABCAM, ab1790 LOT 10158-54-1, Bioss, BSM-52099R LOT AI03046600
Validation	All antibodies were validated by manufacture. Validations studies included western blot analysis, and Immunofluorescence. Specificity of these antibodies was further validated through western blot analysis, see Methods. As H2b represents an essential gene, knockout or knockdown studies were not possible.

Eukaryotic cell lines

Policy information about cell lines and Sex and Gender in Research

Cell line source(s)	Cos7 RRID:CVCL_0224; HCT116 Derivative of RRID:CVCL_RJ11
Authentication	Expression of specific constructs was performed using western blot analysis see Methods
Mycoplasma contamination	All cells used in this study were routinely tested for Mycoplasma using thermal PCR methods as well as imaging of cells labeled with DNA with intercalating dyes. The results of all of these tests indicated that cells were free of contamination.
Commonly misidentified lines (See ICLAC register)	No commonly misidentified cell lines were used in this study.

Plants

Seed stocks	N/A
Novel plant genotypes	N/A
Authentication	N/A
